# Indirect Bypass With Pericranial Transposition for Moyamoya Syndrome in an Infant

**DOI:** 10.7759/cureus.44073

**Published:** 2023-08-24

**Authors:** Rodiyah T Ajala, Anthony Nguyen, Kristopher Lyon, Rabia Qaiser

**Affiliations:** 1 Surgery, Texas A&M School of Medicine, Bryan, USA; 2 Neurosurgery, Baylor Scott & White Medical Center, Temple, USA; 3 Neurological Surgery, Indiana University School of Medicine, Indianapolis, USA

**Keywords:** moyamoya syndrome, moyamoya disease, pediatric neurosurgery, stroke, indirect bypass, encephaloduropericraniosynangiosis

## Abstract

Moyamoya syndrome (MMS) is a progressive disease that can result in debilitating strokes. Surgical revascularization is the mainstay of treatment. Selection of the proper bypass technique depends on the vascular anatomy and location of the hypoperfused cerebral territory. We describe here a case of successful indirect bypass utilizing a pericranial flap as well as dural inversion. A seven-month-old female was transferred from an outside facility to our institution for further evaluation and surgical treatment of MMS. She presented with bilateral brain infarcts worse on the left, with right-sided body weakness. After medical stabilization and hyperhydration, she was taken to the operating room for a left-sided indirect bypass. The superficial temporal artery (STA) was traced utilizing doppler but was determined to be too diminutive for transposition, so the decision was made to proceed with encephalo-duro-pericranio-synangiosis (EDPS). A pericranial graft was successfully affixed to the cortical surface in the hypoperfused middle cerebral artery (MCA) territory, and the dura was inverted. Postoperatively, the patient developed a pseudomeningocele, so a revision surgery was performed. She was discharged shortly after this and returned for encephalo-duro-arterio-synangiosis (EDAS) of the contralateral side. She followed up three months after her initial bypass surgery at age 10 months and was crawling without any focal deficits. She was lost to follow-up thereafter. EDPS is a safe technique for infants with MMS whose STA is too diminutive to be used for bypass surgery. This may be an effective method for indirect bypass in these patients.

## Introduction

Moyamoya disease (MMD) or syndrome (MMS) affects anywhere from 0.086 to 2.3 patients per 100,000 per year worldwide with a bimodal incidence, peaking in childhood and again around middle adulthood [1]. It is a progressive disease, and patients suffer from repeated strokes, accumulating neurologic injuries. The condition preferentially affects the anterior circulation, compromising the territories supplied by the middle cerebral artery (MCA) and anterior cerebral artery (ACA) [2]. The prognosis of MMD is worse in younger patients compared to older patients [3]. While medical management does not reverse the condition, surgical options are available to reperfuse the brain in selective cases [2,4].

The purpose of surgical treatment of MMD/MMS is to restore blood flow to hypoperfused areas of the brain and can be accomplished by either direct or indirect methods [4]. Direct bypass typically involves creating an anastomosis between a branch of the external carotid artery to a cortical arterial branch of an internal carotid artery (ICA), such as a distal MCA branch. The most common of these is the superficial temporal artery (STA) to MCA bypass. Indirect methods involve the transposition of vessels or tissues to place them in close proximity to the cortex. These grafts then undergo angiogenesis and restore blood flow to the at-risk brain. Options for indirect bypass include the STA, dura, temporalis muscle, galea, pericranium, and omentum [4].

The choice of bypass technique depends on various patient factors such as patient’s age, donor vessel size and location of the hypoperfused territory. Younger patient age at the time of bypass is associated with a greater risk of perioperative complications, but postponing surgical treatment may not be appropriate for patients that have sustained multiple infarcts. We describe here a case of indirect bypass in an infant utilizing a pericranial flap along with dural inversion. This report adds our experience to the existing literature describing surgical techniques employed for the treatment of MMD/MMS in infants whose STAs are too diminutive for encephalo-duro-arterio-synangiosis (EDAS) or pial synangiosis.

This article was previously presented as a rapid-fire video presentation at the 2023 Baylor Scott & White Scholars’ Day on May 5, 2023.

## Case presentation

**Figure 1:**
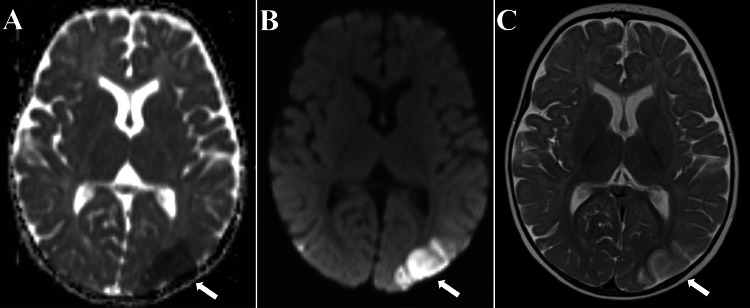
MRI consistent with infarcts of the left parietal lobe and temporo-occipital region with diffusion restriction (white arrows in panels A and B) as evidenced by the apparent diffusion coefficient (A) and diffusion-weighted imaging (B) sequences. This is likewise reflected by hyperintensity (white arrow in panel C) on T2-weighted imaging (C).

**Figure 2:**
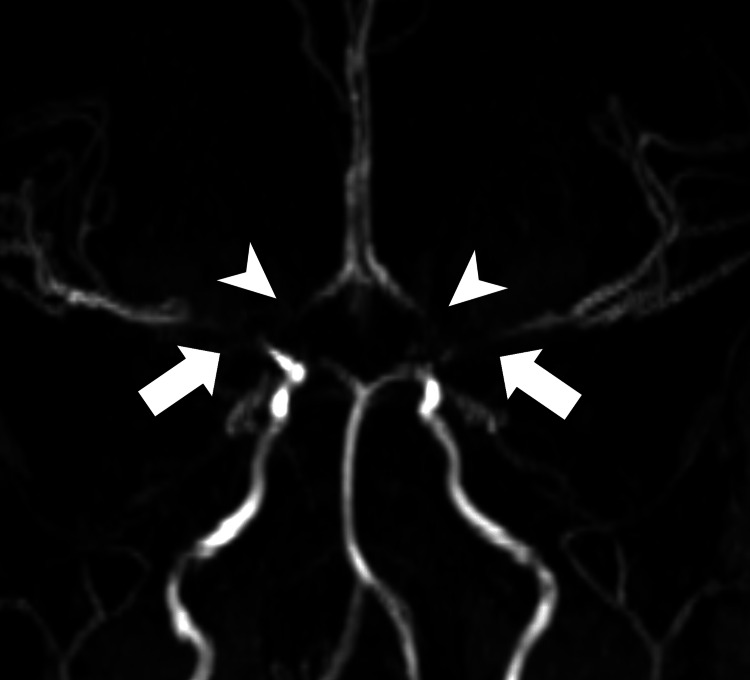
MRA with a standard view of the circle of Willis demonstrating severe stenosis of the distal ICA as well as severe stenosis of the proximal MCAs (white arrows) and proximal ACAs (white arrowheads). MRA: Magnetic resonance angiography; ICA: Internal carotid artery; MCA: Middle cerebral artery; ACA: Anterior cerebral artery

A seven-month-old female with a past medical history of sickle cell trait had been receiving care at an outside hospital system for recurrent strokes. Over the span of a month, the patient had sustained four episodes of strokes. The most recent MRI obtained at the outside hospital system prior to arrival at our institution demonstrated a right frontoparietal infarction and evolving infarctions of the left parietal and left temporo-occipital regions (Figure 1). She had also undergone diagnostic cerebral angiography, which demonstrated stenosis of the terminal ICAs, proximal MCAs, and proximal ACAs bilaterally. Magnetic resonance angiography (MRA) performed following the patient's fourth stroke demonstrated stenosis that had progressed since the conventional catheter angiogram (Figure 2). There was near absence of signal in the distal ICAs and severe loss of signal in the bilateral proximal MCAs and ACAs with the M1 segment of the right MCA being essentially absent. The patient was transferred to our institution for a higher level of care and consideration of revascularization. Upon examination, the patient demonstrated spontaneous movements of all four extremities that were at least antigravity, but her right-sided extremities were stronger than her left. She was started on aspirin 40.5 mg daily and total fluid intake at one-and-a-half times the daily maintenance amount for her weight. 

**Figure 3:**
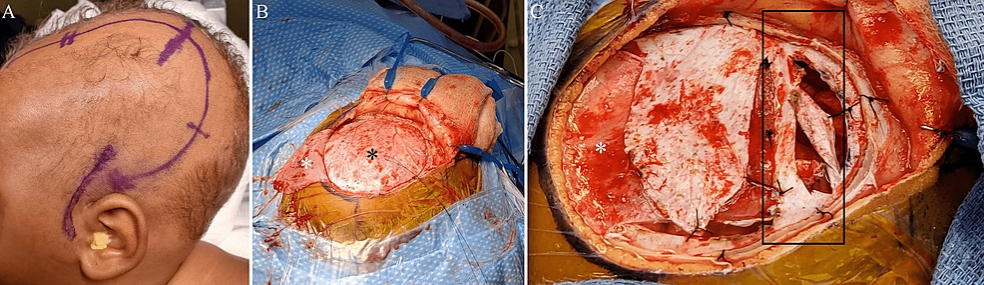
Intraoperative photography demonstrates the reverse question mark incision (A) and the craniotomy (B) with the pericranial flap denoted by the white asterisk and the dura denoted by the black asterisk. The pericranial flap (white asterisk) in place on the pial surface of the brain with the dura inverted (C) and a black rectangle outlines large branches of the middle meningeal artery that have been preserved and inverted.

The patient was taken to the operating room for a left-sided bypass on hospital day four. Using a handheld doppler, the patient's STA was traced. Doppler signal was lost approximately one centimeter above the patient's helix, so an encephalo-duro-arterio-myo-synangiosis (EDAMS) was considered. However, given how posterior the patient's strokes were, the decision was made to instead proceed with indirect bypass by encephalo-duro-pericranio-synangiosis (EDPS). Utilizing a reverse question mark incision that spared the STA, the scalp was dissected down to the pericranium and flapped anteriorly (Figure 3A). The STA was spared in case an indirect or direct bypass would be needed in the future and to preserve blood supply to the surgical wound to promote healing. The pericranial flap was dissected off of the bone leaving a pedicle attached posteriorly, and a 7 cm x 7 cm craniotomy was subsequently performed in a standard fashion, taking care to preserve branches of the middle meningeal artery and other dural arteries (Figure 3B). The dura was opened in a C-shaped fashion with care taken to preserve meningeal blood vessels. The arachnoid was opened widely, the pericranial flap was placed on top of the pial surface of the brain, and dural edges were inverted where possible (Figure 3C). The bone flap was replaced utilizing plates and screws, and the wound was then re-approximated in layers.

The patient developed a pseudomeningocele postoperatively and began to leak from her incision, so she was taken back to the operating room for duraplasty and revision. DuraGen (Integra LifeSciences, Princeton, USA), a collagen-based dural graft, was carefully sutured to the edges of the dura with care taken to avoid compression of the pericranium. The bone flap was re-affixed to the skull, and the patient recovered without recurrence of her pseudomeningocele. 

She returned one month later for staged EDAS of the right side. 10 cm of STA was followed with doppler and outlined. The procedure proceeded in a standard fashion. She was continued on aspirin 40.5 mg and one-and-a-half times her total daily maintenance fluids requirement. She did not develop any neurologic deficits postoperatively and was discharged within a week. She returned to our clinic three months after the initial bypass at the age of 10 months and was doing well. She was crawling and did not have any focal weakness. Her incisions were healing well. She was lost to follow-up thereafter due to moving outside the state, but a clinic note from an outside facility noted that the patient was doing well, moving all extremities, and continuing to improve with therapy.

## Discussion

Bypass is infrequently performed in infants, but our patient’s repeated infarcts indicated the necessity of surgical reperfusion. There are numerous indirect bypass techniques, and most utilize dural inversion. One of the most well-described techniques is the EDAS, which consists of the transposition of the STA to the cortical surface with a wide opening of the arachnoid membrane. Often pial sutures (which results in the procedure being referred to as pial synangiosis) and almost always dural inversion are performed during EDAS [5]. EDAMS and encephalo-duro-myo-arterio-pial-synangiosis (EDMAPS) include transposition of the temporalis muscle as well, and variations of these technique utilize just the temporalis fascia instead of the temporalis muscle [4]. Encephalo-duro-galeo-synangiosis (EDGS) utilizes a galeal or periosteal/pericranial flap along with dural inversion [5]. The multiple burr hole technique involves inverting dural edges into burr holes to allow for indirect bypass [4]. We had planned to proceed with EDAS for the patient’s initial surgery but ultimately had to switch to EDGS due to her diminutive STA.

Revascularization slows the long-term natural progression of MMD/MMS by reducing the number of moyamoya vessels, the risk of cerebral ischemic infarcts, and the risk of intracerebral hemorrhage. This ultimately leads to better intellectual and functional outcomes [6,7]. However, younger age at the time of surgery has been associated with a higher risk of postoperative cerebral infarction [8,9]. It is unclear whether this phenomenon is related to the physiology of younger patients or whether it is a reflection of a more aggressive clinical course of MMD/MMS in younger patients. Hayashi et al. created a scoring system based on MRA findings; a higher MRA score may indicate a greater risk of rapid disease progression, and it has been suggested that patients under the age of four years old with an MRA score of greater than five be considered for surgery within two months of diagnosis [10]. Additionally, although the age of less than three years at the time of surgery was significant in univariable analysis in one study, it was not significant in multivariable analysis, suggesting the possibility of confounders [11]. 

Outcomes data in infants who undergo surgical revascularization is sparse. In a cohort analysis of the multicenter International Pediatric Stroke Study, only 9 out of 174 patients were less than one year of age, and surgical revascularization was performed in 31 out of 174 of the cohort. While age less than one year was not associated with disease recurrence, surgical revascularization was associated with less disease recurrence [12]. The Cho group reports outcomes of a large cohort that includes subsets of patients who undergo only EDAS and patients who undergo EDAS with encephalo-galeo-synangiosis (EGS) [13]. In this 2002 cohort, the youngest age at first operation was seven months. In a subsequent cohort published in 2003 that included only patients who underwent EDAS with EGS, the youngest age at first operation was one year, suggesting the possibility that patients younger than one year of age did not undergo EGS [5]. Storey et al. and Scott et al. reported on the outcomes of cohorts who underwent pial synangiosis, but neither presented outcomes according to patients' age, although Scott et al. mentioned that younger age did not necessarily correspond to poor outcomes [14,15]. In their study, 74% of patients under the age of two years at the time of surgery were completely independent at the time of follow-up more than a year after surgery [15]. Scott et al. reported that long-term functional outcome was related to preoperative functional status, and Storey et al. reported that the presence of preoperative transdural collaterals may be an indication of the patients’ ability to form collaterals from the indirect bypass graft [14,15].

## Conclusions

There is a lack of outcomes data on infants undergoing indirect bypass by utilization of a pericranial flap as the primary pedicle. Our case of a pericranial bypass performed in an infant with MMS provides insight into this. Although infants who require surgical revascularization for MMD/MMS are uncommon, the morbidity of MMD/MMS and repeated strokes may be even more devastating in this group given their younger age and thus potential for greater quality-adjusted life years. However, there are some important limitations of this study to note. Angiographic follow-up data is not available due to the patient’s social constraints, and this is a report of a single case. Larger prospective studies should be conducted to provide better insight into the efficacy of indirect bypass utilizing pericranial flaps in infants whose STAs are too diminutive for EDAS or pial synangiosis.

Bypass options for infants with severe MMD/MMS are more limited as craniotomies cannot be as large, and the STA may be underdeveloped. Indirect bypass with a pericranial graft may be a safe and effective technique for these patients, providing necessary reperfusion to prevent further cerebral infarctions and thus improving long-term neurologic outcomes.
